# Liberia adherence and loss-to-follow-up in HIV and AIDS care and treatment: A retrospective cohort of adolescents and adults from 2016–2019

**DOI:** 10.1371/journal.pgph.0000198

**Published:** 2022-03-23

**Authors:** Keith L. Gray, Murphy Kiazolu, Janjay Jones, Anna Konstantinova, Jethro S. W. Zawolo, Wahdae-Mai Harmon Gray, Naomi F. Walker, Julia T. Garbo, Samretta Caldwell, Michael Odo, Nahid Bhadelia, Jean DeMarco, Laura A. Skrip

**Affiliations:** 1 Health Services, Ministry of Health, Monrovia, Liberia; 2 Evidence Action, Washington, DC, United States of America; 3 College of Health Sciences, University of Liberia, Monrovia, Liberia; 4 Department of Clinical Sciences, Liverpool School of Tropical Medicine, Liverpool, United Kingdom; 5 FHI360, USAID, Monrovia, Liberia; 6 Boston University, Massachusetts, United States of America; 7 University of North Carolina, Chapel Hill, North Carolina, United States of America; Universidad Peruana Cayetano Heredia, PERU

## Abstract

Antiretroviral therapy (ART) is a lifesaving intervention for people living with HIV infection, reducing morbidity and mortality; it is likewise essential to reducing transmission. The “Treat all” strategy recommended by the World Health Organization has dramatically increased ART eligibility and improved access. However, retaining patients on ART has been a major challenge for many national programs in low- and middle-income settings, despite actionable local policies and ambitious targets. To estimate retention of patients along the HIV care cascade in Liberia, and identify factors associated with loss-to-follow-up (LTFU), death, and suboptimal treatment adherence, we conducted a nationwide retrospective cohort study utilizing facility and patient-level records. Patients aged ≥15 years, from 28 facilities who were first registered in HIV care from January 2016 –December 2017 were included. We used Cox proportional hazard models to explore associations between demographic and clinical factors and the outcomes of LTFU and death, and a multinomial logistic regression model to investigate factors associated with suboptimal treatment adherence. Among the 4185 records assessed, 27.4% (n = 1145) were males and the median age of the cohort was 37 (IQR: 30–45) years. At 24 months of follow-up, 41.8% (n = 1751) of patients were LTFU, 6.6% (n = 278) died, 0.5% (n = 21) stopped treatment, 3% (n = 127) transferred to another facility and 47.9% (n = 2008) were retained in care and treatment. The incidence of LTFU was 46.0 (95% CI: 40.8–51.6) per 100 person-years. Relative to patients at WHO clinical stage I at first treatment visit, patients at WHO clinical stage III [adjusted hazard ratio (aHR) 1.59, 95%CI: 1.21–2.09; p <0.001] or IV (aHR 2.41, 95%CI: 1.51–3.84; p <0.001) had increased risk of LTFU; whereas at registration, age category 35–44 (aHR 0.65, 95%CI: 0.44–0.98, p = 0.038) and 45 years and older (aHR 0.60, 95%CI: 0.39–0.93, p = 0.021) had a decreased risk. For death, patients assessed with WHO clinical stage II (aHR 2.35, 95%CI: 1.53–3.61, p<0.001), III (aHR 2.55, 95%CI: 1.75–3.71, p<0.001), and IV (aHR 4.21, 95%CI: 2.57–6.89, p<0.001) had an increased risk, while non-pregnant females (aHR 0.68, 95%CI: 0.51–0.92, p = 0.011) and pregnant females (aHR 0.42, 95%CI: 0.20–0.90, p = 0.026) had a decreased risk when compared to males. Suboptimal adherence was strongly associated with the experience of drug side effects–average adherence [adjusted odds ratio (aOR) 1.45, 95% CI: 1.06–1.99, p = 0.02) and poor adherence (aOR 1.75, 95%CI: 1.11–2.76, p = 0.016), and attending rural facility decreased the odds of average adherence (aOR 0.01, 95%CI: 0.01–0.03, p<0.001) and poor adherence (aOR 0.001, 95%CI: 0.0004–0.003, p<0.001). Loss-to-follow-up and poor adherence remain major challenges to achieving viral suppression targets in Liberia. Over two-fifths of patients engaged with the national HIV program are being lost to follow-up within 2 years of beginning care and treatment. WHO clinical stage III and IV were associated with LTFU while WHO clinical stage II, III and IV were associated with death. Suboptimal adherence was further associated with experience of drug side effects. Active support and close monitoring of patients who have signs of clinical progression and/or drug side effects could improve patient outcomes.

## Introduction

The World Health Organization (WHO) estimated in 2020 that 37.7 million people worldwide were living with HIV (PLHIV), of whom a significant majority of 25.4 million are living in the WHO African region [1]. By the end of 2020, 27.5 million PLHIV had access to antiretroviral therapy (ART) [2]. While some countries in Eastern and Southern Africa have high rates of ART access among PLHIV, retaining patients on ART has been a major challenge for many national programs. In West and Central Africa, where 3.5 million people are accessing ART [3], retention in care by 12 months of follow-up has been reported at 76% [4].

Studies have shown that an adherence rate of no less than 95% is required for treatment success [5], as treatment interruption leads to viral rebound, treatment failure, and drug resistance, and those with clinical AIDS who halt ART tend to die in a short time [6–9]. Several factors have been documented as reasons for interruption, namely loss to follow up (LTFU) among PLHIV. These include undocumented deaths of patients on ART, high risk of LTFU among certain patient subgroups (youths, older age, male), experience with medication side effects, decisions to seek care from a traditional healer or in rural facilities, pregnancy, advanced clinical disease (WHO Stage III and IV), unregistered transfers out of health facilities and distance to health facility [4, 10–17]. Further, patient retention is impacted by health system constraints that affect quality and consistency of care. Such constraints include high attrition rates of trained staff, interrupted supply chain for essential commodities, and differing levels of community interventions to support retention [13].

Countries are now adopting differentiated care models which involves several strategies that can help mitigate health system-level and individual-level barriers to accessing care, enhancing retention and achieving viral suppression [18]. South Africa, Malawi, Uganda, and Mozambique are all high- prevalence HIV countries implementing differentiated models of care. Low-prevalence HIV countries, such as Liberia, have also taken efforts to initiate and retain patients in care.

Since the establishment of its National AIDS and STI Control Program (NACP) in 1986, Liberia has been making strides to reduce the incidence of HIV in the population. Early initiation of ART, irrespective of WHO clinical stage and CD4 cell count, and decentralization of services to primary health facilities have resulted in unprecedented access to treatment services across the country. As part of this expansion of ART, the NACP also adopted the ‘Treat All’ recommendations in WHO’s 2015 Consolidated HIV guidelines [3]. This experience is not unlike many national HIV control programs in Nigeria, Malawi, Mozambique and Zimbabwe, which have variable but slightly higher prevalence than Liberia, ranging from 1.3–11.9% among 15–49 year old [13, 19–21].

The HIV/AIDS context in Liberia—a low-income country in West Africa—reflects a low-level generalized epidemic with an estimated 35000 (29000–43000) people living with HIV (PLHIV), corresponding to an estimated prevalence of 1.1 (0.9–1.4) in adults aged 15–49 years and about 19,000 people on antiretroviral therapy (ART) in 2020 [21]. When compared to rural settings, the prevalence of HIV in urban settings is nearly three times higher, and greater Monrovia where more than a quarter of the Liberian population resides has the highest burden of HIV [22, 23].

Despite ambitious targets and actionable local policies to promote early and accessible ART initiation, retention in care is recognized by healthcare providers as a major challenge to Liberia’s attainment of improved treatment outcomes, yet limited evidence around the care cascade and actual rates of retention exists. It is important for national programs to quantify the prevalence of attrition (*i*.*e*., poor retention) in care and understand context-specific factors associated with it to adequately plan interventions that maximizes efficiency and cost-effectiveness. In Liberia, a previous cross-sectional study examining the impact of the 2014–2015 Ebola virus Disease epidemic on HIV services demonstrated an increase in HIV testing and care enrolment in the population between 2014 and 2015 [24]. However, no study had been conducted to interrogate rates of attrition and identify risk factors associated with different reasons for attrition. In this study, we aimed to examine the care cascade as implemented at ART centers in Liberia. We describe the demographics and clinical characteristics of newly registered patients, and the progression along the HIV care cascade. We estimate retention rates of patients along the HIV care cascade in Liberia, and identify factors associated with LTFU, death, and suboptimal treatment adherence. Outlining the factors associated with attrition and suboptimal adherence among adolescents and adults is expected to inform the allocation of limited resources to develop targeted interventions for better achieving program targets.

## Methods

### Ethical consideration

Ethical approval was obtained from the University of Liberia—Pacific Institute for Research and Evaluation (UL-PIRE), Institutional Review Board (IRB). The IRB waived the requirement for informed consent for use of data from medical records. Data analyzed were anonymized prior to analysis.

### Study design

This retrospective cohort study involved patients ≥15 years, who newly registered for HIV care (*i*.*e*., were recently diagnosed and were receiving HIV care for the first time) at selected treatment facilities across Liberia between 1 January 2016–31 December 2017. Date of diagnosis for patients is typically the same as the date of registration in care. Routinely collected data pertaining to HIV care from medical records for 24 months from the date of registration for HIV care were collected. The study was conducted in strict adherence to the Strengthening the Reporting of Observational Studies in Epidemiology (STROBE) guidelines.

### Study participants and inclusion criteria

The study population included all individuals aged ≥ 15yrs (including pregnant women), who were confirmed HIV-positive and were newly registered at the 28 facilities providing HIV care and treatment services between 1 January 2016 and 31 December 2017. Patients who were restarting ART in the study enrolment period after being lost to follow-up previously were excluded. Any patients whose charts contained inaccurate, incomplete, or missing records on the date of ART initiation were excluded. The records of all eligible patients were included up to 24 months post-registration or until death, LTFU, or transfer out of NACP care, if within 24 months of registration.

### HIV care and treatment services

HIV testing services in Liberia have been made readily accessible and differentiated; testing is available at program-supported voluntary counselling and testing centers, antenatal clinics, health facilities (provider- and self-initiated testing), including in-patient and out-patient departments and blood donation centers across the country [24]. In 2016, the NACP adopted a new “Test and Treat” policy that mandates the immediate initiation of ART for all HIV-positive individuals. Patients who have been screened through various testing strategies are referred to ART clinics for confirmatory testing using a rapid test. Once a person is diagnosed, that person is registered with a unique client identification number and treatment is immediately initiated. Routine follow-up visits for individuals newly registered in care are scheduled more frequently (monthly) to assess compliance and adherence to treatment. All adults (including pregnant and breastfeeding women) are initiated on a first-line ART fixed-dose combination of tenofovir-lamivudine and efavirenz. Patients are generally given ARVs monthly for the first three to six months and based on their adherence, are given three-monthly prescriptions. Patients are also encouraged to visit the clinic on operational days for any medical or psychosocial support needed in between routine HIV care appointments. Routine appointments are scheduled manually on patients’ personal appointment cards (*i*.*e*., “passports”) and in the national program issued paper registers. The timing and frequency are determined based on the number of tablets dispensed, the number remaining from the previous visit, and the general health status of the patient [13]. Patient charts document reasons for visit, any departure from treatment and/or treatment interruption. Factors explaining departure from treatment are coded as stopped ART, transfer (to another facility), LTFU, or death. Some facilities have peer support groups or tracing mechanisms to ascertain these outcomes when possible. Facilities do not have separate death registers, so all outcomes are assigned in the patient’s chart.

### Setting

All health facilities that were registered as HIV service provider sites before 2016 were considered. Purposive sampling was used to select facilities with high PLHIV volume. Urban and rural sites with yearly mean registration rates of ≥150 and ≥50 newly diagnosed individuals, respectively, were selected, comprising twenty-eight facilities from the five sub-regions of Liberia. All but three of the 28 selected facilities are supported by non-governmental (faith-based) organizations. One was supported by both public and private funds. All facilities provide free ART services sponsored by the Global Fund under the auspices of the NACP. Furthermore, all 28 facilities were using a paper-based patient record system at the time of the study.

### Data source and measurement

At each of the 28 selected facilities, data were collected from the Pre- ART and ART registers, patient care and treatment charts (files), viral load and CD4 sample collection and reporting forms, and the NACP’s viral load database. Data were entered from the source forms/registers into an electronic KoBoToolbox data extraction form by data clerks between February–June 2020 (Cambridge, MA, USA).

Pre-ART and ART registers were used to identify eligible patients at each health facility. Patient care and treatment records and viral load and CD4 sample collection and reporting forms were used to collect baseline and follow-up data from the date of enrolment through 24 months for individual patients. Pre-ART patients in the transition period had data collected for both Pre-ART and ART follow-up periods. Time-at-risk observations were analyzed from the time the patient was registered in care and treatment. For all patients, ‘Time-at-risk’ observations for LTFU ended when one of these outcomes was recorded: LTFU, transfer to another facility, death, or retention at 24 months. Data for analyses were extracted from a Microsoft Access (Redmond, Washington, USA) database generated by KoBoToolbox into Microsoft Excel (Microsoft Inc, Redmond, Washington, USA) format.

### Bias

To reduce misclassification bias of LTFU and other key outcomes, we encouraged strict adherence to protocol (variable definitions) as recorded by care providers in patients’ records. Data enumerators worked closely with providers to ascertain outcomes that were ambiguous or discordant or not recorded accurately in ledgers and charts. Additionally, enumerators were not allowed to diagnose and assign outcomes based on the protocol to prevent the introduction of observer bias.

### Study size

The sample was drawn from a total sampling frame or study population of 7136 newly registered HIV-infected individuals in Liberia in 2016 and 2017, according to HMIS data. To account for rare covariates with less strong to very strong signals (*e*.*g*., relative risk of 1.5 to 3, respectively), and assuming an overall 25% event rate, we found that enrolling 1000–4000 individuals gave us about 80% power to detect an association between the covariates and the endpoint. Thus, we targeted a minimum sample size of 4000 patients for inclusion in the sample.

### Variable definitions

The primary outcome variables were LTFU, defined as failure to attend clinic ≥3 months after the patient’s scheduled appointment or missing two consecutive visits without any further contact; death, defined as death from any cause; stopped treatment, defined as discontinuation of treatment for any cause; transferred out, defined as when the patient was transferred formally by a clinician to another ART facility. These outcomes were assessed and documented by the treating clinicians and were not subject to verification. In facilities, patient adherence was assessed by estimating the number of doses missed based on self-report of doses missed, clinic appointment attendance, and medication refill. This was graded on a three-point scale by providers during patient visits—good adherence (missed <3 doses per month), fair adherence (missed 5–8 doses per month), and poor adherence (missed >9 doses per month). To quantify “consistent adherence” to treatment, the following variables were generated based on visits and adherence measured in patients’ records (i) good adherence (still on treatment after 12 months of ART initiation) and adherence degree during the last four visits is “good”; (ii) average adherence (still on treatment after 12 months of ART initiation) and adherence degree for at least 2 of last four visits is “good” and (iii) poor adherence (still on treatment after 12 months of ART initiation) and adherence degree during last four visits was either “poor” or “fair”. Suboptimal adherence was defined as those with average or poor adherence. Other variables of interest among those with viral load testing were virological failure, defined as detectable level of viral load with test results >1000 HIV RNA copies/ml and viral suppression, defined as viral load test results <1000 HIV RNA copies/ml or undetectable (<40 HIV RNA copies/ml).

### Viral suppression estimation for cohort

Liberia has a viral load testing coverage below 50% of people attending antiretroviral therapy. For the cohort, we assumed virologic suppression among patients evaluated for viremia in the cohort to be the same as in those without access to viral load testing services–that is, those tested were representative of the population of HIV-positive individuals on ART in the cohort. We used as numerator, the total number of patients suppressed (<1000 HIV RNA copies/ml) and the total number of patients tested as the denominator. We multiplied the proportion of patients suppressed by the total number of patients retained in care in the sample [25].

### Statistical analysis

Data were checked for completeness and consistency and exported to Stata 16 IC (StataCorp, College Station, TX, USA) and R version 4.0.5 (The R Foundation for Statistical Computing) for cleaning, coding, and analyses. Variables with >25% missingness were excluded from univariable and multivariable analyses. The proportion of participants who had reached each step of the HIV care cascade by 24 months were calculated and graphed. Summary statistics were calculated to provide a description of patients’ demographic, clinical, virologic, and immunologic characteristics, as well as attrition rates from treatment. The frequencies and percentages or medians and interquartile ranges of categorical and continuous demographic, clinical, virologic, and immunologic variables, respectively, were determined for the overall cohort as well as for strata of participants based on sex (males, non-pregnant females, versus pregnant females) and facility location (rural versus urban). Incidence rates and corresponding 95% confidence intervals for LTFU in the cohort were calculated [26]. Next, Cox proportional hazard models were used to calculate the unadjusted and adjusted hazard ratios of LTFU and death against a variety of core predictor variables—age, sex, facility setting, treatment adherence, WHO clinical stage at first visit, TB status at first visit, and drug side effects. Only covariates with statistically significant unadjusted estimates (p<0.05 for at least one level of each categorical variable) were included in the adjusted analyses. The strength of statistical association with LTFU and death was measured by adjusted hazard ratios (AHRs) and associated 95% confidence intervals. Descriptive Kaplan-Meier plots were used to graphically assess LTFU and death across stratifying factors shown to be statistically significantly associated with risk of the outcomes. For treatment adherence, a multinomial logistic regression model was used to characterize associations between adherence levels (good, average, poor) and the same core predictor variables. Only covariates with statistically significant unadjusted estimates were included in the adjusted analyses. Multinomial regression model results were presented as odds ratio (OR) and adjusted odds ratio (aOR) with 95% confidence intervals. Multinomial regression was used for adherence based on the definition and determination of the outcome for patients—which was according to behavior at a specified number of visits and not as a time-to-event metric. For all analyses, statistical significance was set at P<0.05.

### Data quality

Patient records are manually captured in paper registers and charts. Data were extracted from these sources into an electronic format for storage and analyses. External data checks for consistency in data collection and entry were carried out through random selection of 140 study unique identification records from 15 facilities in 8 counties. Also, a comparison of the study data with the District Health Information System 2 (DHIS2) data was done for concordance. The number of HIV cases registered at each facility was compared with the reported number of newly registered HIV cases obtained through the DHIS2 system. It was found that the number of newly registered HIV-positive patients were over reported by 15%. Over reporting was common among females due to double registration during prevention of mother-to-child transmission (PMTCT) program and in the general clinic visits.

## Results

**Fig 1:**
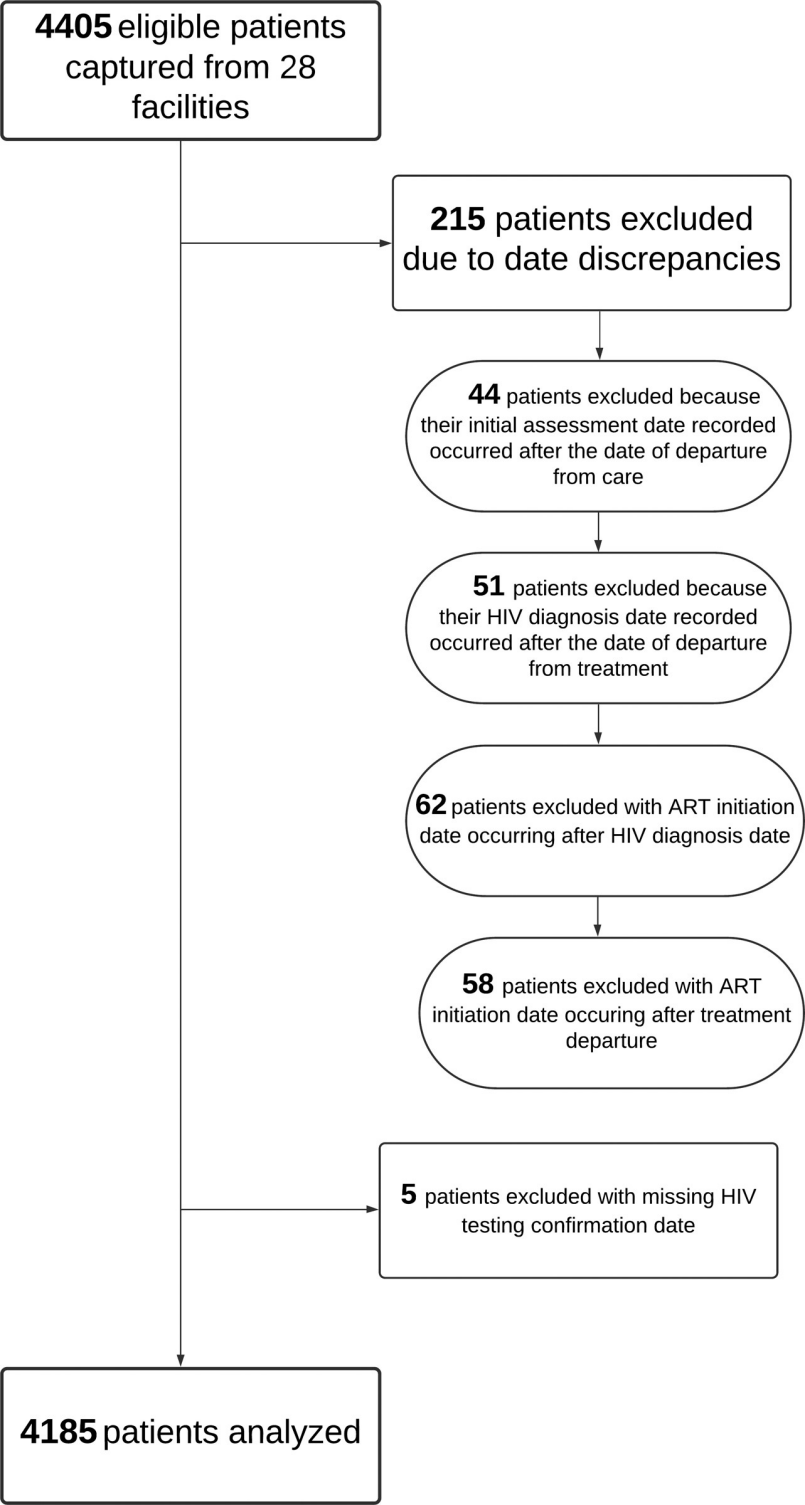
Flow diagram depicting the records of eligible patients reviewed and included in the analyses.

Records from a total of 4185 HIV-positive patients registered in care at 28 health facilities were analyzed; of these patients, 1905 and 2280 were registered in 2016 and 2017 respectively. S1 Table shows the distribution of the records assessed per facility, while Fig 1 provides a flow diagram of how the records of eligible patients were reviewed and assessed for inclusion in the analyses. During the data cleaning process, 44 patients were excluded. In addition, 51 patients were excluded because their recorded HIV diagnosis date occurred after the date of departure from treatment. Both the initial assessment and the confirmatory diagnosis dates should come before the date of attrition, and observations to the contrary were assumed to be due to data quality issues.

### Baseline characteristics of adolescents and adults registered in care and treatment

Table 1 describes the demographic, clinical, and immunological characteristics of the cohort. Of the 4185 patients registered, 1145 (27.4%) were males and 3040 (72.6%) females, with 295 (7.0%) females pregnant at registration in care and treatment. A higher proportion of patients (90.9%, n = 3806) were registered in urban facilities as compared to patients in rural facilities. The median ages at enrollment for males, non-pregnant females and pregnant females were 41 years (IQR: 33–49), 36 years (IQR: 29–43) and 31 years (IQR: 26–38), respectively. One third (33.1%) of the patients registered were in the age category of 35–44 years and females accounted for the majority, 72.6%. Two-fifths (40.1%) of males registered were 45 years and older.

**Table 1:** Characterization of demographical, clinical, and programmatic parameters of the cohort at baseline and outcomes at 24 months of follow-up.

		By Gender			By Facility Location	
	Overall	Male	Female (Not Pregnant)	Female (Pregnant When HIV Confirmed)	Rural	Urban
	(n = 4,185)	(n = 1,145)	(n = 2,745)	(n = 295)	(n = 379)	(n = 3,806)
**A. Gender**						
*Male*	1,145	-	-	-	81	1,064
* *	(27.4%)	-	-	-	(21.4%)	(29.0%)
*Female (Not Pregnant)*	2,745				287	2315
	(65.5%)				(75.7%)	(63.2%)
*Female (Pregnant)*	295	-	-	-	11	284
* *	(7.0%)	-	-	-	(2.9%)	(7.8%)
**B. Facility Location**						
*Rural*	379	81	287	11	-	-
	(9.1%)	(7.1%)	(10.5%)	(3.7%)		
*Urban*	3,806	1064	2458	284	-	-
	(90.9%)	(92.9%)	(89.5%)	(96.3%)		
**C. Age**						
*Median Age Upon registration in Care*	37	41	36	31	35	37
* *	(IQR: 30–45)	(IQR: 33–49)	(IQR: 29–43)	(IQR: 26–38)	(IQR: 29–44)	(IQR: 30–45)
*Aged 15–24*	391	52	268	63	47	344
	(9.3%)	(4.5%)	(10.3%)	(21.4%)	(12.4%)	(9.0%)
*Aged 25–34*	1,260	249	851	116	122	1,138
	(30.1%)	(21.7%)	(32.7%)	(39.3%)	(32.2%)	(29.9%)
*Aged 35–44*	1,384	385	861	93	107	1,277
	(33.1%)	(33.6%)	(33.1%)	(31.5%)	(28.2%)	(33.6%)
*Aged 45+*	1,150	459	622	23	103	1,047
	(27.5%)	(40.1%)	(23.9%)	(7.8%)	(27.2%)	(27.5%)
**D. CD4**						
*Has CD4 Tested at One of First Two Visits*	369	125	216	28	0	369
* *	(8.8%)	(10.9%)	(7.9%)	(9.5%)	(0%)	(8.8%)
*Median CD4 Count (at First Visit for Patients Tested)*	345	387	309	459	-	348
* *	(IQR: 160–550)	(IQR: 176–539)	(IQR: 145–475)	(IQR: 195–655)	-	(IQR: 163–552)
**E. WHO Clinical Stage (at initial assessment)**						
*WHO Stage 1*	1,979	501	1,249	197	213	1,766
* *	(55.7%)	(50.7%)	(57.9%)	(71.9%)	(59.7%)	(55.2%)
*WHO Stage 2*	570	159	337	37	68	502
* *	(16.0%)	(16.1%)	(15.6%)	(13.5%)	(19.0%)	(15.7%)
*WHO Stage 3*	845	271	480	31	72	773
* *	(23.8%)	(27.4%)	(22.3%)	(11.3%)	(20.2%)	(24.2%)
*WHO Stage 4*	161	58	90	9	4	157
* *	(4.5%)	(5.9%)	(4.2%)	(3.3%)	(1.1%)	(4.9%)
**F. Departure From Treatment**						
*Departed from Treatment (Any reason)*	2,177	623	1,291	126	180	1,997
* *	(52.0%)	(54.4%)	(49.6%)	(42.7%)	(47.5%)	(52.5%)
Lost to Follow-Up**	1,751	481	1,043	109	128	1,623
* *	(80.4%)	(77.2%)	(80.8%)	(86.5%)	(71.1%)	(81.3%)
Transferred**	127	35	80	5	5	122
* *	(5.8%)	(5.6%)	(6.2%)	(4.0%)	(2.8%)	(6.1%)
Stopped Treatment**	21	6	13	2	7	14
* *	(1.0%)	(1.0%)	(1.0%)	(1.6%)	(3.9%)	(0.7%)
Death**	278	101	155	10	40	238
* *	(12.8%)	(16.2%)	(12.0%)	(7.9%)	(22.2%)	(11.9%)
*Median Time to LTFU From Initial Assessment (Months)*	9.3	9.7	9.1	10.0	6.0	10.2
* *	(IQR: 4.4–17.3)	(IQR: 4.5–16.9)	(IQR: 4.3–17.5)	(IQR: 5.0–18.9)	(IQR: 4.0–9.4)	(IQR: 4.6–18.0)
*Median Time to LTFU From ART Initiation (Months)*	9.0	10.1	8.7	11.3	8.5	9.5
* *	(IQR: 5.2–16.9)	(IQR: 5.8–14.1)	(IQR: 4.4–17.1)	(IQR: 5.6–18.4)	(IQR: 5.0–9.1)	(IQR: 5.2–17.2)
*Median Time to Death From Initial Assessment (Months)*	5.4	4.4	5.9	6.2	5.6	5.4
* *	(IQR: 2.1–15.5)	(IQR: 1.5–13.5)	(IQR: 2.3–16.3)	(IQR: 3.0–10.8)	(IQR: 1.7–14.1)	(IQR: 2.2–15.6)
*Median Time to Death From ART Initiation (Months)*	3.8	11.6	3.8	6.2	6.1	6.0
* *	(IQR: 1.4–7.4)	(IQR: 6.2–15.7)	(IQR: 2.8–4.9)	(IQR: 4.6–8.5)	(IQR: 2.5–12.0)	(IQR: 3.4–8.8)

** % is out of total Departed from Treatment.

Among patients enrolling in care, less than a tenth (8.8%) of the cohort had CD4+ cell count assessed at either of the first two visits, with no tests performed in a rural facility. For those who had been tested within the first two visits, the median CD4+ count was 345 cells/mm^3^ (IQR: 160–550). At care and treatment initiation, more than one-half (55.7%) of the patients registered were categorized under WHO clinical stage I at initial assessment, irrespective of facility location.

Of the 2177 (52%) patients who departed from treatment during follow-up, 1751 (80.2%) were categorized as LTFU, with the highest proportion (86.5%) occurring among women who were pregnant at registration. The median times to LTFU from initial assessment and ART initiation were 9.3 (IQR: 4.4–17.3) months and 9 (IQR: 5.2–16.9) months, respectively. Deaths accounted for 12.8% (n = 278) of departure from care, with a higher proportion (36.3%, 101/278) occurring among males relative to among non-pregnant or pregnant females. Among the deaths, the median time for those initiating ART was shorter (median: 3.8 months, IQR 1.4–7.4) months compared to patients just initiating care services (median: 5.4 months, IQR: 2.1–15.5%).

### The HIV care cascade

**Fig 2:**
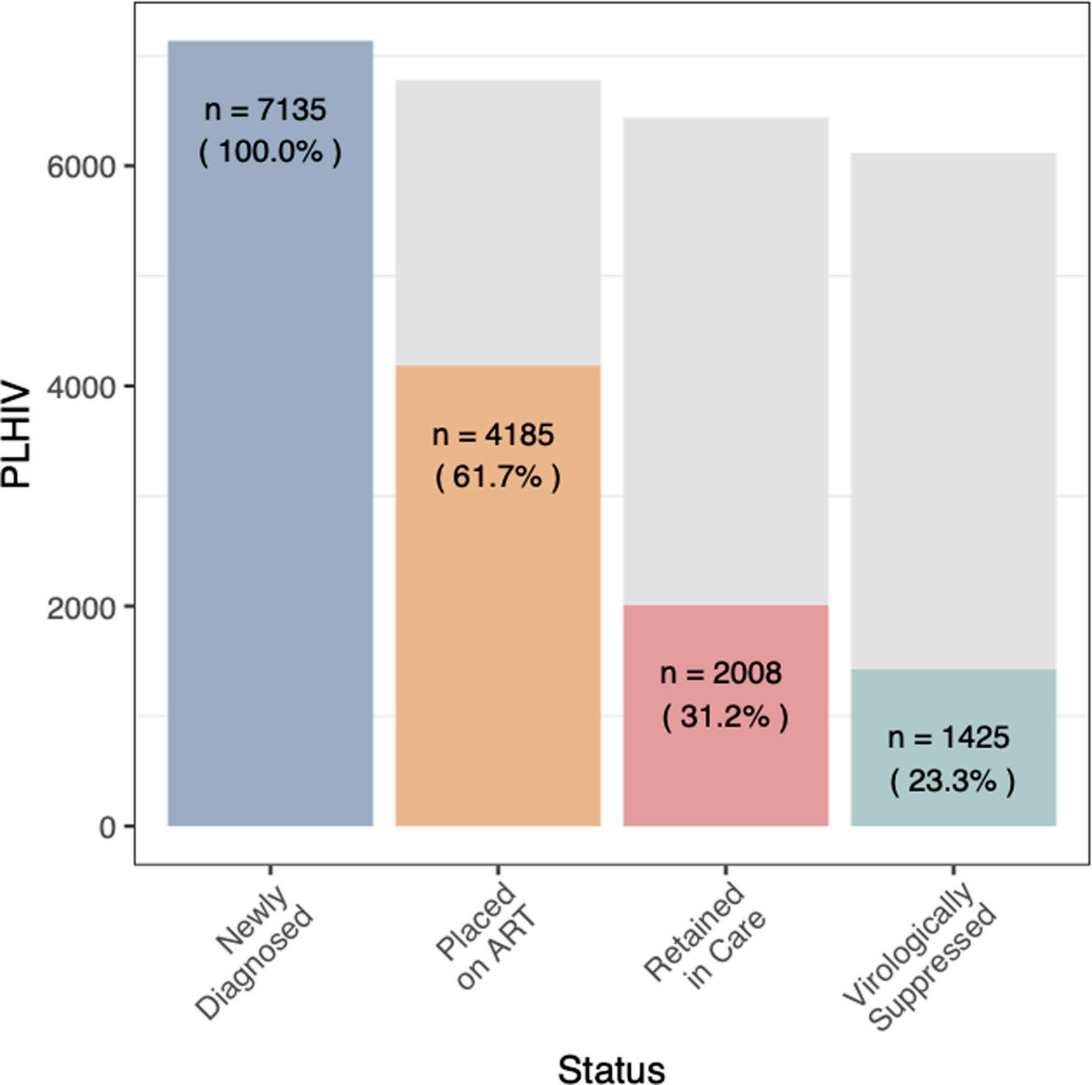
HIV care cascade during 24 months of follow up for individuals registered in care during 2016 and 2017 across 28 facilities in Liberia. Blue bar- represents the total number of people diagnosed with HIV, 15 years and older, across the 28 health facilities as reported by the District Health Information System 2 (DHIS2); amber–represents the total number of eligible patients registered for care and treatment services at the 28 facilities; red–represents retention and the key outcomes of patients in care and treatment after 24 months of follow up; green–represents the number of patients evaluated for viremia among patients retained in care; gray–represents the gap along each stage of the care continuum.

Fig 2 illustrates the care cascade for the cohort. In 2016 and 2017, the number of PLHIV identified at the 28 facilities by DHIS2 records was 7136. Of the 7136 patients identified, 4405 records of adolescents and adults registered into care and treatment were assessed. Of the 4185 eligible patients, after 24 months of follow-up, 41.8% (n = 1751) of patients were LTFU, 6.6% (n = 278) died, 0.5% (n = 21) stopped treatment, 3% (n = 127) transferred to another facility and 47.9% (n = 2008) were retained in care and treatment.

### Incidence of LTFU

The 4185 patients registered for care and treatment generated 5625.3 person-years of follow-up. The incidence rate of LTFU was 46.0 (95% CI: 40.8–51.6) per 100 person years.

### Factors associated with loss-to-follow-up

**Fig 3:**
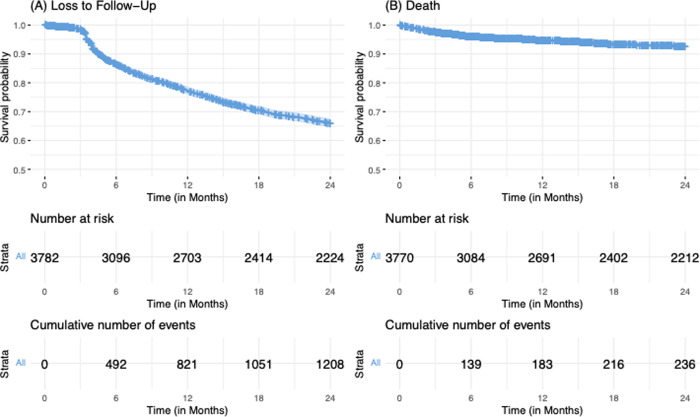
Kaplan-Meier probability of loss-to-follow-up and death among patients who were registered in 2016 and 2017 and followed for 24 months.

**Fig 4:**
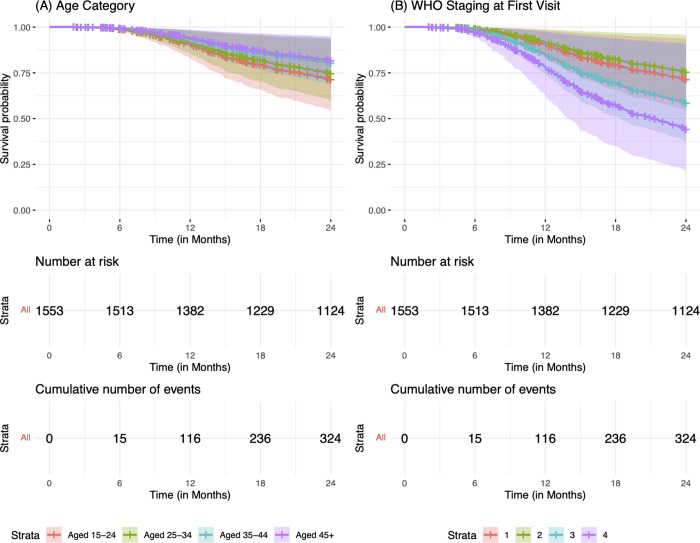
Kaplan-Meier estimate of loss-to-follow-up per age category and WHO clinical stage classification at registration in care and treatment among patients followed for 24 months.

In the unadjusted analysis, average adherence, WHO stages II, III and IV, and treatment side effects were associated with increased risk of LTFU, relative to people with good adherence, baseline clinical staging at WHO stage I, and no treatment side effects (Table 2). On the contrary, older age groups (35–44 years and 45 years and older) and being a pregnant female were associated with decreased risk, relative to younger age groups (15–24 year) and being male. Relative to risk of LTFU among patients enrolled in care during January 1-March 31, 2016, registration into care during all subsequent quarters was associated with reduced risk of LTFU. However, after adjustment (specifically, in a multivariable model including age, sex, consistent adherence, WHO clinical stage, treatment side effects, time period of registration, and site), patients classified as WHO stage III (AHR: 1.59, 95%CI: 1.21–2.09; p<0.001) and IV (AHR: 2.41, 95%CI: 1.51–3.84; p<0.001) had a higher risk of LTFU as compared to patients classified as WHO stage I. Also, adults in the age groups 35–44 years (AHR: 0.65, 95%CI: 0.44–9.84; p<0.038) and 45 years and above (AHR: 0.60, 95%CI: 0.39–0.93; p<0.021) had 35% and 40% risk reduction, respectively, relative to individuals aged 15–24 years. Figs 3 and 4 demonstrate the overall probability of LTFU and the probability of LTFU by age category and WHO clinical stage, respectively.

**Table 2:** Estimates of factors associated with loss-to-follow-up among adolescents and adults in care and treatment.

Covariates	Unadjusted		Adjusted‡	
	Hazard ratio (95% Confidence Interval)	p value	Hazard ratio (95% Confidence Interval)	p value
**Age**	n = 3,782		n = 1,553	
15–24	Ref			
25–34	0.85 (0.71, 1.03)	p = 0.104	0.87 (0.58, 1.29)	p = 0.485
35–44	0.62 (0.51, 0.76)	p<0.001**	0.65 (0.44, 0.98)	p = 0.038*
45+	0.57 (0.47, 0.70)	p<0.001**	0.60 (0.39, 0.93)	p = 0.021*
**Sex Category**	n = 3,678		n = 1,553	
Male	Ref			
Female (Not Pregnant)	0.92 (0.81, 1.04)	p = 0.183	0.82 (0.64, 1.04)	p = 0.106
Female (Pregnant)	0.59 (0.45, 0.79)	p<0.001**	0.97 (0.49, 1.89)	p = 0.921
**Setting**	n = 3,782			
Urban	Ref			
Rural	1.12 (0.89, 0.93)	p = 0.220		
**Consistent Adherence**	n = 1,997		n = 1,553	
Good	Ref			
Average	1.26 (1.01, 1.57)	p = 0.039*	1.14 (0.88, 1.49)	p = 0.319
Poor	1.17 (0.89, 1.55)	p = 0.271	1.09 (0.76, 1.55)	p = 0.640
**WHO Stage (at First Visit)**	n = 3,209		n = 1,553	
1	Ref			
2	1.23 (1.03, 1.46)	p = 0.023*	0.84 (0.57, 1.23)	p = 0.358
3	1.70 (1.48, 1.95)	p<0.001**	1.59 (1.21, 2.09)	p<0.001**
4	1.89 (1.46, 2.46)	p<0.001**	2.41 (1.51, 3.84)	p<0.001**
**TB Symptoms (at First Visit)**	n = 3,056			
No Symptoms	Ref			
TB Suspected or Confirmed	1.15 (0.97, 1.36)	p = 0.109		
**Drug Side Effects**	n = 2,826		n = 1,553	
No Side Effects	Ref			
Had Side Effects	1.24 (1.09, 1.42)	p = 0.002*	1.18 (0.89, 1.58)	p = 0.255
**Time Period (3-month increment)**	n = 3,781		n = 1,553	
January 1-March 31, 2016	Ref			
April 1-June 30, 2016	0.76 (0.61, 0.95)	p = 0.003*	1.08 (0.64, 1.79)	p = 0.783
July 1-September 30, 2016	0.72 (0.58, 0.89)	p = 0.002*	1.16 (0.72, 1.87)	p = 0.532
October 1-December 31, 2016	0.70 (0.58, 0.89)	p<0.001**	1.09 (0.65, 1.82)	p = 0.742
January 1-March 31, 2017	0.61 (0.49, 0.77)	p<0.001**	1.30 (0.79, 2.14)	p = 0.309
April 1-June 30, 2017	0.63 (0.50, 0.78)	p<0.001**	0.91 (0.55, 1.51)	p = 0.709
July 1-September 30, 2017	0.55 (0.43, 0.69)	p<0.001**	0.85 (0.49, 1.49)	p = 0.580
October 1- December 31, 2017	0.51 (0.40, 0.65)	p<0.001**	0.76 (0.43, 1.33)	p = 0.336

‡ Adjusted for site.

** Significant at p<0.001.

* Significant at p<0.05.

Not significant in unadjusted analysis.

### Factors associated with death

**Fig 5:**
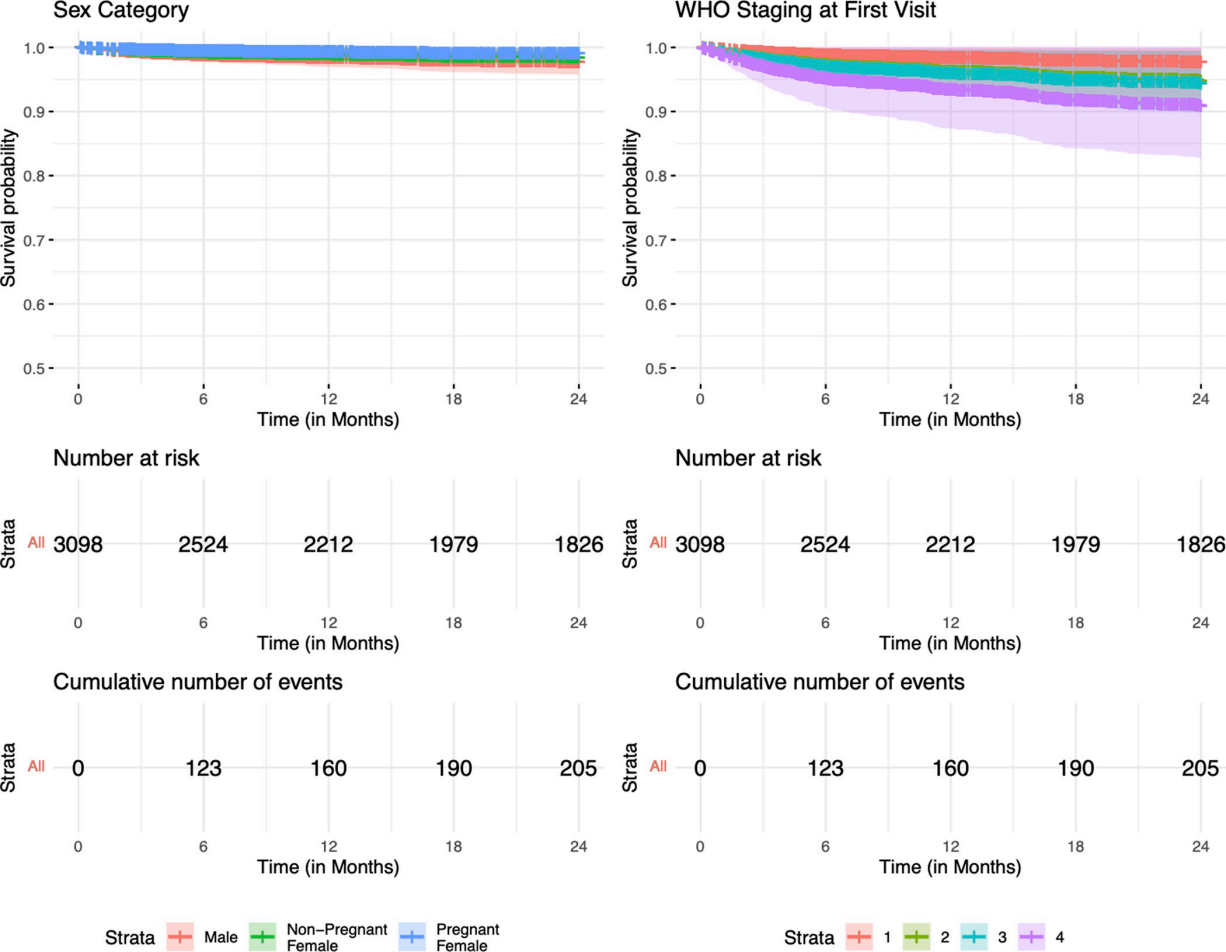
Kaplan-Meier survivor estimate per age category and WHO clinical stage classification at registration in care and treatment among patients followed for 24 months.

Table 3 shows unadjusted and adjusted model estimates of factors associated with death among patients registered in care and treatment. Unadjusted estimates showed that WHO clinical stages II, III, and IV and accessing care in a rural facility were associated with increased risk of death, relative to clinical staging at WHO stage 1 and accessing care in an urban facility, respectively. In contrast, being a non-pregnant or pregnant female was associated with a decreased risk of death as compared to being male. After adjustment in a multivariable model including sex, WHO staging, rural versus urban setting, time period and site, advanced clinical disease at enrollment with WHO stages II (aHR: 2.35, 95%CI: 1.53–3.61; p<0.001), III (aHR: 2.55, 95%CI: 1.75–3.71; p<0.001) and IV (aHR: 4.21, 95%CI: 2.57–6.89; p<0.001) was associated with significantly higher risk of death when compared to disease staged at WHO stage I upon enrollment in care. On the other hand, being a non-pregnant female or a pregnant-female reduced the risk of death by 32% (aHR: 0.68, 95%CI: 0.51–0.92; p = 0.011) and 58% (aHR: 0.42, 95%CI: 0.20–0.90; p = 0.026), respectively, as compared to being male. Figs 4 and 5 illustrate the cohort’s overall probability of death and the probability of death per sex category and WHO clinical stage, respectively.

**Table 3:** Estimates of factors associated with death among adolescents and adults in care and treatment.

Covariates	Unadjusted		Adjusted‡		
	Hazard ratio (95% Confidence Interval)	p value	Hazard ratio (95%Confidence Interval)	p value	
**Age**	n = 3,770				
15–24	Ref				
25–34	1.22 (0.69, 2.14)	p = 0.490			
35–44	1.46 (0.85, 2.53)	p = 0.172			
45+	1.39 (0.80, 2.43)	p = 0.247			
**Sex Category**	n = 3,666		n = 3,098		
Male	Ref				
Female (Not Pregnant)	0.64 (0.48, 0.83)	p = 0.001*	0.68 (0.51, 0.92)		p = 0.011*
Female (Pregnant)	0.36 (0.17, 0.74)	p = 0.005*	0.42 (0.20, 0.90)		p = 0.026*
**Setting**	n = 3,770		n = 3,098		
Urban	Ref				
Rural	1.69 (1.18, 2.42)	p = 0.004*	0.37 (0.12, 1.10)		p = 0.073
**Consistent Adherence**	n = 1,992				
Good	Ref				
Average	0.73 (0.44, 1.19)	p = 0.205			
Poor	0.51 (0.24, 1.06)	p = 0.071			
**WHO Stage (at First Visit)**	n = 3,197		n = 3,098		
1	Ref				
2	1.80 (1.24, 2.61)	p = 0.002*	2.35 (1.53, 3.61)		p<0.001**
3	1.84 (1.32, 2.56)	p<0.001**	2.55 (1.75, 3.71)		p<0.001**
4	4.80 (3.14, 7.35)	p<0.001**	4.21 (2.57, 6.89)		p<0.001**
**TB Symptoms (at First Visit)**	n = 3,044				
No Symptoms	Ref				
TB Suspected or Confirmed	1.14 (0.77, 1.67)	p = 0.514			
**Drug Side Effects**	n = 2,817				
No Side Effects	Ref				
Had Side Effects	1.07 (0.79, 1.44)	p = 0.674			
**Time Period (3-month increment)**	n = 3,769				
January 1-March 31, 2016	Ref				
April 1-June 30, 2016	1.76 (1.00, 3.11)	p = 0.049*	1.44 (0.80, 2.58)		p = 0.223
July 1-September 30, 2016	1.11 (061, 2.01)	p = 0.731	0.82 (0.45, 1.52)		p = 0.537
October 1-December 31, 2016	1.23 (0.67, 2.26)	p = 0.503	0.92 (0.49, 1.75)		p = 0.800
January 1-March 31, 2017	1.21 (0.67, 2.17)	p = 0.531	0.95 (0.50, 1.83)		p = 0.884
April 1-June 30, 2017	1.17 (0.65, 2.09)	p = 0.603	1.06 (0.56, 1.97)		p = 0.865
July 1-September 30, 2017	1.17 (0.64, 2.13)	p = 0.618	1.16 (0.61, 2.18)		p = 0.653
October 1-December 31, 2017	1.08 (0.59, 1.99)	p = 0.797	1.03 (0.55, 1.94)		p = 0.917

‡ Adjusted for site.

** Significant at p<0.001.

* Significant at p<0.05.

Not significant in unadjusted analysis.

### Factors associated with suboptimal adherence

Analysis of suboptimal adherence included, age, sex, facility setting, WHO clinical stage, TB assessment and drugs side effects as covariates (Table 4). Unadjusted estimates showed an increased odds of suboptimal adherence (average and/or poor) among pregnant women relative to men, among patients classified with WHO stages II, III, or IV relative to those classified with WHO stage I, patients with suspected or confirmed TB relative to no signs of TB, and among patients reporting drug side effects relative to no side effects. The odds of average adherence versus good adherence was estimated to be half of among patients attending a rural facility relative to patients attending an urban facility. Conversely, adjusted estimates showed an increased odds of suboptimal adherence among patients reporting drug side effects compared to patients without side effects. Further, the odds of suboptimal adherence remained significantly reduced among patients registered in a rural facility as compared to an urban facility.

**Table 4:** Estimates of factors associated with suboptimal adherence among adolescents and adults in care and treatment.

Covariates	Unadjusted				Adjusted ‡			
	Average		Poor		Average		Poor	
	Odds ratio (95% Confidence Interval)	P value	Odds ratio (95% Confidence Interval)	P value	Odds ratio (95% Confidence Interval)	P value	Odds ratio (95% Confidence Interval)	P value
**Age**	n = 2089							
15–24	Ref							
25–34	1.05 (0.72, 1.54)	0.807	0.89 (0.57, 1.38)	0.599				
35–44	0.96 (0.66, 1.40)	0.837	0.70 (0.45, 1.10)	0.121				
45+	1.10 (0.75, 1.62)	0.618	0.69 (0.44, 1.10)	0.124				
**Sex Category**	n = 2061				n = 1548			
Male	Ref							
Female (Not Pregnant)	0.87 (0.69, 1.09)	0.233	0.87 (0.65, 1.17)	0.354	0.87 (0.64, 1.18)	0.372	1.03 (0.66, 1.62)	0.882
Female (Pregnant)	1.36 (0.91, 2.02)	0.135	1.66 (1.03, 2.67)	0.037*	0.76 (0.40, 1.46)	0.412	1.19 (0.49, 2.92)	0.702
**Setting**	n = 2089				n = 1548			
Urban	Ref							
Rural	0.51 (0.35, 0.73)	<0.001**	1.01 (0.69, 1.49)	0.941	0.01 (0.01, 0.03)	<0.001**	0.001 (0.0004, 0.003)	<0.001**
**WHO Stage (at First Visit)**	n = 1945				n = 1548			
1	Ref							
2	1.15 (0.86, 1.54)	0.348	1.46 (1.02, 2.08)	0.038*	0.71 (0.47, 1.08)	0.111	0.85 (0.48, 1.50)	0.573
3	1.40 (1.09, 1.81)	0.008*	1.51 (1.09, 2.07)	0.012*	0.85 (0.57, 1.25)	0.409	0.80 (0.46, 1.39)	0.434
4	1.73 (1.06, 2.81)	0.028*	2.03 (1.13, 3.64)	0.018*	1.29 (0.61, 2.75)	0.510	1.85 (0.68, 5.05)	0.230
**TB Symptoms (at First Visit)**	n = 1894				n = 1548			
No Symptoms	Ref							
TB Suspected or Confirmed	1.52 (1.14, 2.01)	0.004*	1.69 (1.18, 2.42)	0.004*	1.08 (0.72, 1.63)	0.713	1.56 (0.90, 2.69)	0.113
**Drug Side Effects**	n = 1794				n = 1548			
No Side Effects	Ref							
Had Side Effects	2.50 (2.00, 3.11)	<0.001**	3.65 (2.72, 4.91)	<0.001**	1.45 (1.06, 1.99)	0.02*	1.75 (1.11, 2.76)	0.016*
**Time Period (3-month increment)**	n = 2089				n = 1548			
January 1-March 31, 2016	Ref							
April 1-June 30, 2016	1.20 (0.76, 1.88)	0.434	0.87 (0.52, 1.46)	0.592	1.52 (0.79, 2.91)	0.208	1.84 (0.78, 4.31)	0.161
July 1-September 30, 2016	1.42 (0.93, 2.18)	0.104	0.87 (0.53, 1.43)	0.582	1.42 (0.77, 2.62)	0.263	0.97 (0.41, 2.30)	0.944
October 1-December 31, 2016	0.94 (0.59, 1.49)	0.797	0.87 (0.52, 1.45)	0.597	1.57 (0.79, 3.10)	0.197	1.79 (0.73, 4.41)	0.204
January 1-March 31, 2017	0.60 (0.38, 0.93)	0.002*	0.21 (0.12, 0.39)	<0.001**	0.69 (0.38, 1.28)	0.245	0.46 (0.19, 1.14)	0.094
April 1-June 30, 2017	0.69 (0.44, 1.06)	0.089	0.39 (0.23, 0.66)	<0.001**	0.79 (0.45, 1.45)	0.455	0.74 (0.32, 1.71)	0.480
July 1-September 30, 2017	0.66 (0.42, 1.05)	0.078	0.38 (0.22, 0.66)	<0.001**	0.83 (0.44, 1.55)	0.550	0.60 (0.24, 1.52)	0.281
October 1-December 31, 2017	0.89 (0.57, 1.38)	0.603	0.73 (0.44, 1.19)	0.207	1.70 (0.91, 3.17)	0.094	2.44 (1.06, 5.64)	0.036*

‡ Adjusted for site.

** Significant at p<0.001

* Significant at p<0.05.

Not significant in unadjusted analysis.

## Discussion

We found that the rate of LTFU among people living with HIV after 2 years on ART was high. Being classified with advanced WHO clinical stage at the initial assessment was associated with increased risks of LTFU and death among the cohort, relative to classification at WHO stage I during enrollment in care. Compared to those without side effects, patients experiencing side effects had an increased likelihood of sub-optimal ART adherence. In contrast, there was a decreased odds of suboptimal adherence among patients attending rural facilities as compared to those attending urban facilities. Our study found no significant differences between males and females (non-pregnant or pregnant) being LTFU and sub-optimal adherence, although both pregnant and non-pregnant women were at decreased risk of death relative to male patients. Overall, the findings highlight patient characteristics that challenge efforts to retain patients in care and treatment.

Timely initiation of patients on ART through the ‘test and treat” policy aims to enroll patients early in the course of the natural history of HIV infection, to reduce morbidity, mortality and onward transmission through viral suppression. As a motivation behind adoption of the ‘Treat All’ policy, the NACP recognized that there was low retention of patients in care and treatment and suboptimal adherence to ART, despite limited quantification as the extent of the problem. The overall LTFU among those registered in care in 2016 and 2017 and followed for 24 months was 41.8%. Our results were similar to previous findings from Liberia that demonstrated 46% retention after 2 years of follow-up [27]. A retrospective study assessing LTFU among pre-ART and ART patients found 26% of those initiating ART were lost to follow up; while the rates of LTFU among pre-ART and ART clients followed for 24 months estimated LTFU at 48 and 26 per 100 person-years, respectively [13]. When compared to these findings, it had been hypothesized that the present study would demonstrate an improvement in retention of patients in care by increasing early access to ART and improving treatment outcomes, as per the “Treat All” policy. Further study in Liberia and elsewhere is needed to evaluate the policy and challenges around implementation, as there is paucity of recent evidence examining the impact of the “Treat All” policy on national HIV programs.

Maintaining high retention of recipients of care is critical to the national program’s success in ensuring viral suppression and better quality of life. Our study demonstrated a retention of 48% after 24 months of follow-up. These findings are markedly lower than the 71% retention rate for a similar period of follow-up after ART initiation seen in other low and middle income countries [27]. This further emphasizes the need for newer retention strategies to improve patient outcomes in comparison to increasing ART access. In addition, studies [4, 28] assessing retention in ART programmes in Western and Southern Africa observed higher retentions, 76.1%, and 79.2%, respectively, compared to our findings. This may be due to the definition of LTFU used. These studies [4, 58] defined LTFU as ‘patients who had not been in contact with the HIV clinics for at least 6 months or 180 days as compared to our definition of failure to attend visits for ≥3 months.

Several contextual factors could help to explain the finding of no change in LTFU rates before and after the ‘Treat All’ policy change in Liberia. Unlike countries such as South Africa which has a higher burden of HIV than Liberia but also has widespread as well as targeted efforts to reduce stigma [29, 30], Liberia still lags in combatting stigmatization around HIV and sero-status disclosure. Moreover, other countries like South Africa, Zimbabwe, Malawi and Mozambique use differentiated models of care delivery that help to increase retention and adherence to ART [4, 18]. Such approaches have not been adopted in Liberia. Retention is enhanced when PLHIV are offered treatment with consideration of individual preferences and context. Furthermore, in part, the higher rate of LTFU in our study may be due to data quality issues, with records indicating patients who have missed a scheduled appointment being misclassified as LTFU. Efforts to harmonize data across facilities to account for transfers or data classification errors warrant investment. Also, for LTFU, the deaths of patients with advanced disease in communities or faith-based healing centers may have been inappropriately classified by care providers at health facilities and thus may have contributed to the higher rate.

We also found higher risk of LTFU among patients who were classified as WHO clinical stage III or IV at their initial assessment, as compared to patients who were classified as WHO clinical stage I. These findings were corroborated by previous studies assessing rates and predictors of LTFU and outcomes of patients receiving ART in Zimbabwe, Malawi, Ethiopia, and South Africa [4, 13, 31, 32]. The results of our study also offer additional evidence that remedies for drug side effects may encourage increased retention in care [11]. Currently the NACP has transitioned to a tenofovir-lamivudine-dolutegravir first-line regimen which is associated with reduced side effects and may show improvements in future analyses. Further, patient education on drugs administered and coping strategies, and the availability of other essential medications to mitigate the side effect are important for adherence.

On the contrary, our study also found that among the cohort, age groups, 35–44 years and 45 years and above had significantly lower risks of LTFU than the age group 15–24 years. These results were consistent with findings in Zimbabwe and Tanzania [19, 33]. One possibility could be that youths, identified predominantly through outreach testing activities, may be in denial at the time of diagnosis, due to the lack of opportunistic infections that increase morbidity. Another possibility is that the NACP retention strategies are mainly targeted towards adults in care, rather than youths. In West Africa, evidence has shown that HIV status disclosure improves retention in care among adolescence [34].

Inconsistent with our findings, evidence from Malawi, Haiti and Nigeria has shown that non-pregnant females [35] and pregnant females [36] initiating universal lifelong ART were at high risk of attrition [37]. One reason for the contrasting results may be the heterogeneity of methods and definition of LTFU. For instance, one study [35] used a cross-sectional design while assessing 13 years of patient records; whereas, the other [37] defined LTFU as 60 days after a missed appointment and used a 6-month assessment period. Another reason may be the counseling pregnant women received at facilities on the benefits of treatment adherence and to a lesser extent, the mandatory screening for HIV among pregnant women (and the testing record to be presented) at subsequent visits makes treatment more acceptable in Liberia.

It is of interest that our overall mortality rate (6.6%) was similar to findings of Bernard et al, who reported a mortality rate of 5.9% across nine West African Countries (excluding Liberia) over 3-year periods between 2006 and 2016 [38]. Our findings have also demonstrated a strong association between advanced HIV disease and mortality, in accordance with results in Ivory Coast [39] and in countries in other regions—Ethiopia and Mozambique [20, 40, 41].

Furthermore, among the cohort, crude estimates did not show a strong independent association of mortality with patients suspected or confirmed TB. This contrasted with other findings in Africa [42, 43]. There may be several reasons for this. Liberia is one of the World Health Organization’s 30 high burden countries for TB and HIV-associated TB. Strong coordination between the national HIV and TB programs has fostered screening for the two infections at many health facilities using an integrated service approach. Patients in care and treatment are also initiated on isoniazid preventive treatment (IPT) for TB, mitigating the risk of progression of latent TB to active TB. Further, the immediate initiation of ART in people living with HIV, because of the “Test and Treat” policy may have also played a role. Current evidence suggests that no administration of ART in people with HIV increased mortality and the mortality risk for TB-HIV co-infected was least with early ART initiation [39, 44]. Additionally, by 2017, many patients had been switched to the tenofovir-lamivudine-efavirenz first-line regimen which had lesser adverse effects than the zidovudine-lamivudine-nevirapine regimen, thus improving adherence and survival. Because of the higher HIV prevalence in urban areas, rural facilities tend to be less congested, and patients have easier access to service providers.

Optimal adherence to ART is important to achieve and sustain viral suppression. Because in Liberia adherence is mainly self-reported and is graded on a 3-point scale ranging from poor to good, and varies from one clinic appointment to the next, we estimated the risk of sub-optimal adherence for patients on ART for at least a year. A comparative analysis of ART adherence of cohorts in Africa and Asia have shown that low- and lower-middle-income countries had a higher risk of suboptimal adherence, with key determinants being male, younger age, concomitant medication, and attending public facility [45]. Our findings did not show significant associations of sub-optimal adherence with age, sex, pregnancy and HIV clinical stage categorization. Also, crude estimates showed that patients suspected or confirmed with TB had an increased likelihood. Adjusted analysis however demonstrated that TB had no profound impact on treatment adherence, consistent with a systematic review of patients receiving ART in sub-Saharan Africa and a retrospective study in South Africa, respectively [46, 47]. There may be several reasons for these differences. One reason may have been due to how adherence was assessed. Clinicians in Liberia rely mostly on patients’ self-reports with less frequent physical pill verification, as opposed to a structured questionnaire assessing adherence. Another reason may have been due to the perceived health status of patients. Patients who have been on treatment may feel healthy and become inconsistent with treatment until the frequency of opportunistic infections increases. While pill count during scheduled appointments seems an objective measure, patients may get rid of, or stash away pills not taken and return to the facility with the anticipated number of pills to give an impression of good adherence [47–49]. Additionally, evidence have demonstrated the possibility of plasma virological suppression with sub-optimal ART adherence [47, 50, 51].

Conversely, our study demonstrated a significantly decreased risk of suboptimal adherence among patients attending rural facilities, unlike findings in Nigeria and Uganda [52, 53]. These findings differ due to the proxy measure–ART provided > 60 days and not being classified as LTFU, transferred, nor deceased–for non-adherence in lieu of pill count used in one study [53] and an ART adherence level measured on the proportion of pill count and limited to adolescents [52].

There have been several causes of low ART adherence in Africa [54]. One key factor for nonadherence is the adverse effects of drugs [46, 55, 56], consistent with our findings. Evidence has shown that patients are less likely to adhere to treatment if they were experiencing adverse effects of drugs [46, 57]. With Liberia transitioning to a new first-line regimen of tenofovir-lamivudine-dolutegravir, the reduced side effects and once a day dosing regimen should improve adherence. Another factor is the perceived level of confidentiality and the dispensing of ARVs only to health facilities. Studies have shown that adherence improved with perceived better confidentiality and differentiated ART delivery model that promotes patient-centered care through decentralized mechanisms [58, 59]. Moreover, structured early monitoring and support with continuous assessment can yield long-term optimal adherence [47].

The strengths of our study include the use of patient-level and facility-level datasets across multiple facilities in both rural and urban settings within the 5 subregions of Liberia. Also, our study followed patient records over a 2-year period to describe characteristics of patients newly registered in care and treatment services and provide operational outcomes of the HIV care cascade. Our study also used a large sample size with adequate statistical power to generate estimates of the incidence and factors associated with LTFU, death, and suboptimal adherence to ART.

The limitations of our study include the exclusion of records with missing or inaccurately captured registration dates, thus introducing a selection bias. We found considerable missing data additionally from appointment visits. For example, data on CD4 measurements, viral load, opportunistic infections, weight, and height were more than 25% missing and could not be assessed in the analysis. LTFU may have been overestimated by providers due to the lack of resources to track patients, leading to the possibility that patients classified as LTFU may have self-referred or transferred to other facilities to continue ART, or died. There is no gold standard to measure medication adherence. Providers relied on self-reports (which is susceptible to recall or social desirability bias) and pill count (which does not provide evidence of actual ingestion of the drug). A more robust qualitative approach, using a multi-method (self-report questionnaire, visual analog scale, and simplified medication adherence questionnaire is needed to further examine adherence. Finally, the purposive sampling method selected patients from higher burden clinics for inclusion and therefore, the findings cannot necessarily be extrapolated to lower burden settings in the country.

## Conclusion

In conclusion, our results demonstrate that retention in care remains a challenge despite a “test and treat” policy and free access to ART and patients’ adherence monitoring. It is critical to better understand why patients are not retained in care and treatment and devise interventions to reduce loss of patients, to reduce mortality and morbidity from HIV and control HIV transmission in the region. These results highlight the urgent need for innovative solutions to transform outcomes of current HIV care services.

## Supporting information

S1 Checklist Items checklist that was included in the cohort report. (DOCX)

S1 Table Location, sample size distribution and relative fraction of records assessed at the 28 study sites per year of registration. (DOCX)

S2 Table Analysis of viral load coverage from January 2016 through December 2019 for the 28 study sites. (DOCX)
