# Supplementary material for: Liberia adherence and loss-to-follow-up in HIV and AIDS care and treatment: A retrospective cohort of adolescents and adults from 2016–2019
Source: PLOS Glob Public Health. 2022 Mar 23;2(3):e0000198. doi: 10.1371/journal.pgph.0000198 (PMC10021315; doi:10.1371/journal.pgph.0000198)
Supplement: S1 Table — (DOCX) [file pgph.0000198.s002.docx]

|  | | |  |  | |
| --- | --- | --- | --- | --- | --- |
| Region | County | Health Facility |  | Sample Size | Fraction of overall sample |
| North Western | Bomi | Liberia Government Hospital (Bomi) |  | 137 | 3.3% |
| North Western | Bomi | Jenneh Number 3 Health Center |  | 21 | 0.5% |
| North Central | Bong | Charles B. Dunbar Hospital |  | 109 | 2.6% |
| North Central | Bong | Phebe Hospital |  | 137 | 3.3% |
| North Central | Lofa | Foya Borma Hospital |  | 51 | 1.2% |
| North Central | Lofa | Tellewoyan Memorial Hospital |  | 85 | 2.0% |
| North Central | Lofa | Kolahun Hospital |  | 31 | 0.7% |
| North Central | Nimba | Bahn Health Center |  | 60 | 1.4% |
| North Central | Nimba | Ganta United Methodist Hospital |  | 188 | 4.5% |
| North Central | Nimba | Sacleapea Comprehensive Health Center |  | 83 | 2.0% |
| North Central | Nimba | Karnplay Health Center |  | 55 | 1.3% |
| South Central | Grand Bassa | Liberia Government Hospital |  | 56 | 1.3% |
| South Central | Margibi | C.H. Rennie Hospital |  | 176 | 4.2% |
| South Central | Margibi | Du-Side Hospital |  | 71 | 1.7% |
| South Central | Montserrado | Clara Town Health Center |  | 187 | 4.5% |
| South Central | Montserrado | Duport Road Health Center |  | 301 | 7.2% |
| South Central | Montserrado | ELWA Hospital |  | 364 | 8.7% |
| South Central | Montserrado | James N. Davies Memorial Hospital |  | 123 | 2.9% |
| South Central | Montserrado | John F. Kennedy Health Center |  | 625 | 14.9% |
| South Central | Montserrado | National TB & Leprosy Hospital |  | 235 | 5.6% |
| South Central | Montserrado | Redemption Hospital |  | 718 | 17.1% |
| South Central | Montserrado | St. Joseph's Catholic Hospital |  | 77 | 1.8% |
| South Central | Montserrado | Star Of The Sea Health Center |  | 56 | 1.3% |
| South Eastern A | Grand Gedeh | Martha Tubman Memorial Hospital |  | 45 | 1.1% |
| South Eastern A | Grand Gedeh | Konobo Health Center |  | 7 | 0.2% |
| South Eastern B | Maryland | J.J. Dossen Hospital |  | 57 | 1.4% |
| South Eastern B | Maryland | Pleebo Health Center |  | 60 | 1.4% |
| South Eastern B | Maryland | St. Francis Clinic |  | 70 | 1.7% |
