# Supplementary material for: Liberia adherence and loss-to-follow-up in HIV and AIDS care and treatment: A retrospective cohort of adolescents and adults from 2016–2019
Source: PLOS Glob Public Health. 2022 Mar 23;2(3):e0000198. doi: 10.1371/journal.pgph.0000198 (PMC10021315; doi:10.1371/journal.pgph.0000198)
Supplement: S2 Table — (DOCX) [file pgph.0000198.s003.docx]

|  | **2016** | **2017** | **2018** | **2019** |
| --- | --- | --- | --- | --- |
| **A. Number of HIV Care Visits (Total)** |  |  |  |  |
| *Overall* | 7,660 | 15,863 | 11,486 | 4,944 |
| *Males* | 2,033 | 4,022 | 2,911 | 1,189 |
| *Females* | 5,627 | 11,841 | 8,575 | 3,755 |
| *Rural Facility* | 591 | 1,269 | 949 | 480 |
| *Urban Facility* | 7,069 | 14,594 | 10,537 | 4,464 |
| **B. Tested for Viremia (Total and as % of Visits)** |  |  |  |  |
| *Overall* | 99 | 112 | 70 | 86 |
|  | 1.3% | 0.7% | 0.6% | 1.7% |
| *Males* | 21 | 28 | 14 | 16 |
|  | 1.0% | 0.7% | 0.5% | 1.3% |
| *Females* | 78 | 84 | 56 | 70 |
|  | 1.4% | 0.7% | 0.7% | 1.9% |
| *Rural Facility* | 9 | 18 | 1 | 1 |
|  | 1.5% | 1.4% | 0.1% | 0.2% |
| *Urban Facility* | 90 | 94 | 69 | 85 |
|  | 1.3% | 0.6% | 0.7% | 1.9% |
| **C. Achieved Viral Suppression (Total and as % of Tested)** |  |  |  |  |
| *Overall* | 68 | 77 | 49 | 66 |
|  | 68.7% | 68.8% | 70.0% | 76.7% |
| *Males* | 17 | 19 | 10 | 14 |
|  | 81.0% | 67.9% | 71.4% | 87.5% |
| *Females* | 51 | 58 | 39 | 52 |
|  | 65.4% | 69.0% | 69.6% | 74.3% |
| *Rural Facility* | 7 | 13 | 1 | 0 |
|  | 77.8% | 72.2% | 100.0% | 0.0% |
| *Urban Facility* | 61 | 64 | 48 | 66 |
|  | 67.8% | 68.1% | 69.6% | 77.6% |
